# Isolation and Functional Characterization of Erythrofibrase: An Alfa-Fibrinogenase Enzyme from *Trimeresurus erythrurus* Venom of North-East India

**DOI:** 10.3390/toxins16040201

**Published:** 2024-04-22

**Authors:** Susmita Thakur, Rafika Yasmin, Anita Malhotra, Hmar Tlawmte Lalremsanga, Vishal Santra, Surajit Giri, Robin Doley

**Affiliations:** 1 Molecular Toxinology Laboratory, Department of Molecular Biology and Biotechnology, Tezpur University, Tezpur 784028, Assam, India; sushmitat92@gmail.com (S.T.); rafikayasmin@gmail.com (R.Y.); 2 Molecular Ecology and Evolution at Bangor, School of Environmental and Natural Sciences, Bangor University, Bangor LL57 2UW, UK; a.malhotra@bangor.ac.uk; 3 Developmental Biology and Herpetology Laboratory, Department of Zoology, Mizoram University, Aizawl 796004, Mizoram, India; htlrsa@yahoo.co.in; 4 Society for Nature Conservation, Research and Community Engagement (CONCERN), Nalikul 712407, West Bengal, India; vishal.herp9@gmail.com; 5 Captive and Field Herpetology, 13 Hirfron, Anglesey LL65 1YU, UK; 6 Shree Sainath Surgical and Maternity Hospital, Valsad 396050, Gujrat, India; 7 Demow Government Community Health Centre, Raichai, Konwar Dihingia Gaon, Sivasagar 785662, Assam, India; drsurajitgiri@outlook.com

**Keywords:** green pit viper, *Trimeresurus erythrurus*, antivenom, fibrinogenolytic protein, fibrinogenase, chromatography

## Abstract

Green pit viper bites induce mild toxicity with painful local swelling, blistering, cellulitis, necrosis, ecchymosis and consumptive coagulopathy. Several bite cases of green pit vipers have been reported in several south-east Asian countries including the north-eastern region of India. The present study describes isolation and characterization of a haemostatically active protein from *Trimeresurus erythrurus* venom responsible for coagulopathy. Using a two-step chromatographic method, a snake venom serine protease erythrofibrase was purified to homogeneity. SDS-PAGE of erythrofibrase showed a single band of ~30 kDa in both reducing and non-reducing conditions. The primary structure of erythrofibrase was determined by ESI LC-MS/MS, and the partial sequence obtained showed 77% sequence similarity with other snake venom thrombin-like enzymes (SVTLEs). The partial sequence obtained had the typical 12 conserved cysteine residues, as well as the active site residues (His57, Asp102 and Ser195). Functionally, erythrofibrase showed direct fibrinogenolytic activity by degrading the Aα chain of bovine fibrinogen at a slow rate, which might be responsible for causing hypofibrinogenemia and incoagulable blood for several days in envenomated patients. Moreover, the inability of Indian polyvalent antivenom (manufactured by Premium Serum Pvt. Ltd., Maharashtra, India) to neutralize the thrombin-like and plasmin-like activity of erythrofibrase can be correlated with the clinical inefficacy of antivenom therapy. This is the first study reporting an α-fibrinogenase enzyme erythrofibrase from *T. erythrurus* venom, which is crucial for the pathophysiological manifestations observed in envenomated victims.

## 1. Introduction

Haemostasis is a highly regulated vital physiological process of the body which functions to maintain the normal blood flow, preventing excessive blood loss resulting from vascular injury [1]. The haemostatic system includes a repertoire of various cellular and sub-cellular moieties, ions, enzymes and pro-enzymes which, upon proteolytic cleavage, activate a cascade of reactions facilitating the formation of a clot, thereby preventing blood loss following an injury. Any glitch in this vital array of inter-dependent reactions leads to pathophysiological conditions like thrombosis and haemorrhage. 

The haemostatic system is the most susceptible target for various proteins/peptides of Viperidae snake venoms. In particular, the coagulation cascade, platelet aggregation pathway and fibrinolytic system mark the foremost targets for the majority of venom toxins which show either anticoagulant or procoagulant effect in patients [2,3]. Moreover, a particular type of pseudo-procoagulant effect is observed mostly during pit viper envenomation, which is characterized by the abundance of snake venom thrombin-like enzymes (SVTLEs), a subgroup of serine proteases. The function of SVTLEs is analogous to thrombin, targeting the fibrin polymerization step of the coagulation cascade to form a thrombus. However, unlike thrombin, SVTLEs cannot activate factor XIII, as a result of which the clot formed is not stabilized and is thereby easily removed by the fibrinolytic system [4,5]. Some of the SVTLEs are known to be fibrinogenolytic enzymes, which cleave the fibrinogen monomers, rendering them unclottable by thrombin. This characteristic of SVTLEs instigates a defibrinating syndrome in patients, leading to a situation commonly known as venom-induced consumptive coagulopathy (VICC). As a result of the pseudo-procoagulant property, SVTLEs are used as a defibrinogenating agent for treating patients with some thrombo-embolic syndromes like ischemic stroke and peripheral artery diseases [6,7]. A few examples include ancrod from *Calloselasma rhodostoma*, batroxobin from *Bothrops atrox*, and reptilase from *Bothrops jararaca* [2,6,8]. 

Green pit vipers (*Trimeresurus* and relatives) are one of the leading causes of venomous bites in several Asian countries, including the north-eastern region of India [9,10,11,12,13]. Indian-green-pit-viper-envenomated patients are typically characterized by painful local swelling, blistering, cellulitis, necrosis, ecchymosis and consumptive coagulopathy [9]. Such venom-induced consumptive coagulopathy is frequently accompanied by low platelet count, hypofibrinogenemia and defibrination syndrome, causing prolonged coagulopathy persisting up to 10–20 days [11,14]. The abundance of phospholipases A_2_s (PLA_2_s), snake venom serine proteases (SVSPs) and snaclecs in the venom can be correlated with the prolonged coagulopathy observed in the victims [15]. Further, painful edema, blistering, necrosis, haemorrhage and platelet alteration can be attributed to snake venom metalloproteases (SVMPs), L-amino acid oxidases and disintegrins [16]. Previous studies have reported the presence of these protein families in the venom of Indian spot-tailed pit viper *Trimeresurus erythrurus* [17]. Further, the clinical ineffectiveness of commercially available polyvalent antivenom in reversing the prolonged coagulopathy has been reported previously [14]. However, the major toxic components of Indian green pit vipers that causes incoagulable blood in envenomated patients have not been identified. Investigations undertaking a search for major venom toxins might lead to a better understanding of the pharmacological profile of the venom and its immuno-reactivity, as well as the identification of potent bioactive molecules, which could be explored further as promising therapeutic agents. Therefore, the present study has been undertaken to identify and characterize a haemostatically active protein from the venom of Indian *Trimeresurus erythrurus* with a special emphasis on snake venom thrombin-like enzymes. 

## 2. Results

### 2.1. Purification of Erythrofibrase from Trimeresurus erythrurus Venom

**Figure 1:**
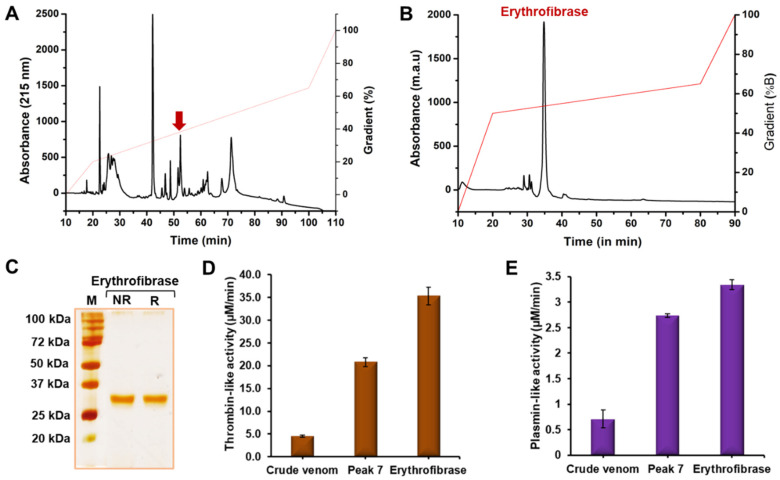
Purification of erythrofibrase. (**A**) Rp-HPLC of crude venom of *Trimeresurus erythrurus*. Crude venom (1 mg) was loaded on Symmetry C18 column, and fractionation was carried on with a linear gradient of 20–65% acetonitrile containing 0.1% TFA. Peak 7 possessing thrombin-like and plasmin-like activity is indicated by arrow mark. (**B**) Re-chromatography of Peak 7. Peak 7 (50 µg) was loaded on Acclaim C18 column, and fractionation was carried out with a gradient of 45–65%. (**C**) 12.5% tris-glycine SDS-PAGE of erythrofibrase. M represents standard protein ladder. NR and R represents non-reducing and reducing conditions, respectively. (**D**) Thrombin-like activity of erythrofibrase. (**E**) Plasmin-like activity of erythrofibrase. Each data point represents mean ± SD of three independent experiments.

Purification of erythrofibrase from *Trimeresurus erythrurus* venom was performed using a two-step chromatographic approach. Crude venom was fractionated into 10 peaks of varying intensities using Rp-HPLC, and erythrofibrase was purified from Peak 7 based on thrombin-like and plasmin-like activity (Figure 1A,B). The homogeneity of the purified fraction was confirmed by SDS-PAGE which showed a single prominent band at ~30 kDa in both a non-reducing and reducing condition (Figure 1C). Further, erythrofibrase showed both thrombin-like and plasmin-like activity significantly more than Peak 7 and crude venom, suggesting an increase in specific enzyme activity with the increase in purity (Figure 1D,E). 

**Figure 2:**
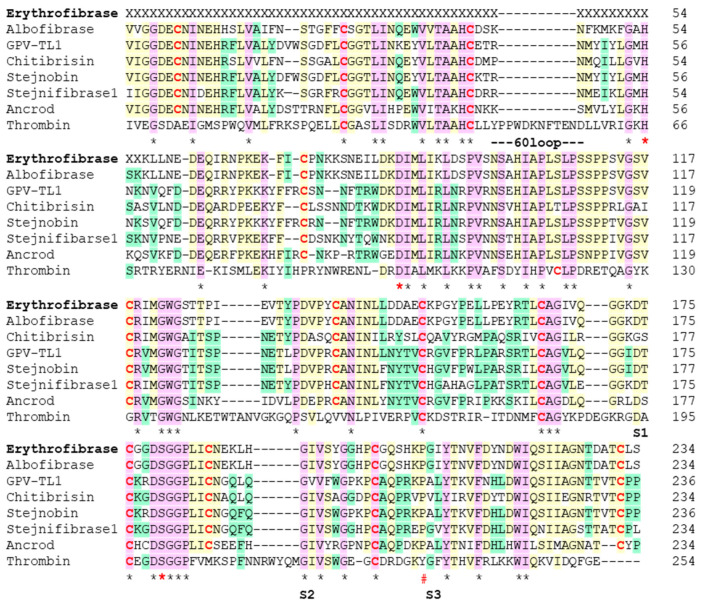
Multiple sequence alignment of erythrofibrase with serine proteases. Albofibrase (accession no. P0CJ41) from *T. albolabris*, GPV-TL1 (accession no. A7LAC6) from *Trimeresurus albolabris*, chitibrisin (accession no. P0DJF6) from *T. albolabris*, stejnobin (accession no. Q8AY81) from *V. stejnegeri*, stejnofibrase 1 (accession no. AAN52348.1) from *V. stejnegeri*. ancrod (accession no. P47797.1) from *Calloselasma rhodostoma* and heavy chain of human α-thrombin (accession no. 494462). Identical residues were marked in pink colour along with asterisk (*). Yellow and green colours represent residues with ≥75% and ≥50% homology, respectively. X represents unidentified residues and — marks the positions with no corresponding amino acid residue. The residues of catalytic triad, i.e., Histidine, Aspartate and Serine, were highlighted with red colour asterisk (*). Cysteine residues were highlighted in red bold font. S1, S2 and S3 represents subsites which bind to substate. # represents Na^2+^ binding site of thrombin.

In-solution trypsin digestion and LC-MS/MS of erythrofibrase resulted in the identification of 18 peptide fragments of varying length. A detailed summary of identified peptide fragments of erythrofibrase is listed in Supplementary Table S1. Based on the sequence homology of peptide fragments, erythrofibrase was identified to be homologous to alpha-fibrinogenase albofibrase (Accession no. P0CJ41.1) with a molecular weight of 28 kDa. The partial amino acid sequence obtained was aligned with the reported sequence of albofibrase, and it was observed that all the amino acid residues from position 81 to 258 of albofibrase were identified in erythrofibrase (Figure 2).

### 2.2. Sequence Alignment of Erythrofibrase

Multiple sequence alignment of amino acid residues of erythrofibrase with other serine proteases show high sequence similarity with at least 30 consensus residues (Figure 2). The most homologous protein, albofibrase, showed 27.5% similarity with thrombin and about 76.9% similarity with other SVTLEs (GPV-TL1, stejnobin and ancrod). Further, about 64.1% similarity was observed between albofibrase and Stejnifibrase 1, an alpha-fibrinogenase enzyme isolated from *Viridovipera stejnegeri*. The active site residues His57, Asp102 and Ser195 (Chymotrypsin numbering system), forming the catalytic triad, were conserved in all the sequences. The major difference was observed in the number of cysteine residues responsible for the formation of disulfide bonds. Unlike 7 cysteine residues in thrombin, albofibrase possesses 12 cysteine residues which were conserved in all the SVTLEs (Figure 2). The subsites S1, S2 and S3 containing Asp189, Gly216 and Gyl/Ala 226 represents the additional residues which bind to the substrate. Na^2+^ binding Tyr255 of thrombin is replaced by Pro255 in all SVTLEs. Also, all the SVTLEs have a C-terminal extension of five amino acid residues, which is absent in thrombin. 

### 2.3. Functional Characterization of Erythrofibrase

**Figure 3:**
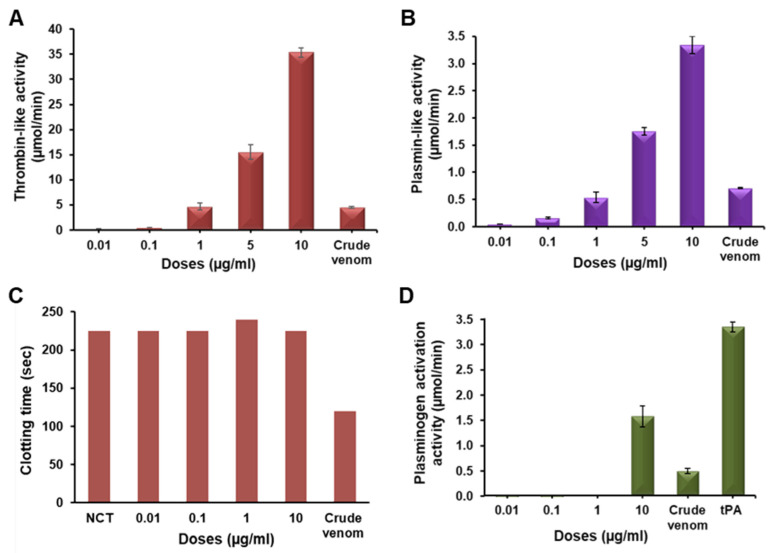
Functional characterization of erythrofibrase. (**A**) Dose-dependent thrombin-like activity (**B**) Dose-dependent plasmin-like activity (**C**) Recalcification Time (RT). NCT—normal clotting time. (**D**) Plasminogen activation assay. tPA (10 nM) was taken as positive control. Crude venom was taken as additional control for each set of experiments.

Erythrofibrase showed amidolytic activity on both S2238 and S2251 specific for thrombin and plasmin in a dose-dependent manner, inferring the thrombin-like and plasmin-like activity of erythrofibrase (Figure 3A,B). Moreover, the clotting time of platelet-poor plasma (PPP), incubated with erythrofibrase, was found to be similar to the normal clotting time (Figure 3C), indicating that erythrofibrase does not affect the calcium-induced coagulation of PPP. Further, at a dose of 10 µg/mL, erythrofibrase showed 1.56 ± 0.207 µmol/min plasminogen activation, which was significantly higher than the plasminogen activation of crude venom, i.e., 0.48 ± 0.06 µmol/min (Figure 3D). 

### 2.4. Effect of Erythrofibrase on Fibrinogenolytic and Fibrinolytic System

**Figure 4:**
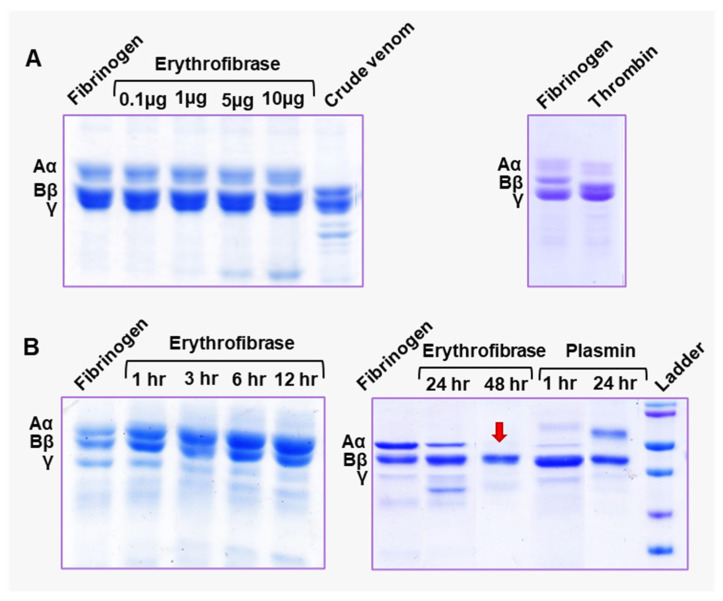
Fibrinogenolytic activity of erythrofibrase. (**A**) Dose-dependent (**B**) Time-dependent fibrinogenolytic activity. Thrombin (10 u/mL) and Plasmin (1 µg) were taken as positive control, and Fibrinogen without any treatment was taken as negative control. Ladder represents standard protein markers from molecular weight range 20–72 kDa. Red colour arrow marks the empty position in gel created by degradation of Aα band of fibrinogen by erythrofibrase.

Since erythrofibrase shows thrombin-like activity, its effect on the natural substrate of thrombin, i.e., fibrinogen, was assessed by a fibrinogenolytic activity assay. The SDS-PAGE profile of untreated fibrinogen shows three prominent bands, Aα, Bβ and γ (Figure 4). Upon treatment with thrombin (1 h of incubation), a mild shift in the position of Aα and Bβ band was observed, pointing towards a reduction in chain length. (Figure 4A). Markedly, fibrinogen treated with 10 µg of erythrofibrase showed partial degradation of the Aα band after 24 h of incubation, suggesting mild fibrinogenolytic activity of erythrofibrase (Figure 4B). Extended incubation till 48 h showed complete degradation of the Aα chain (Figure 4B), which was similar to that of plasmin-treated fibrinogen. 

**Figure 5:**
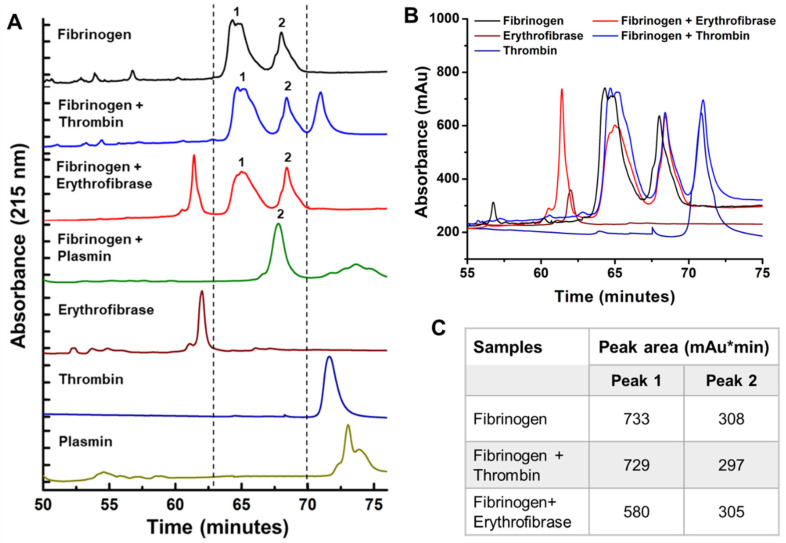
Chromatographic profile showing fibrinogenolytic activity of erythrofibrase. (**A**) Comparative Rp-HPLC profile of treated and untreated samples. Bovine fibrinogen was incubated with erythrofibrase, thrombin and plasmin for 1 h. Post-incubation, cleavage pattern of fibrinogen was analyzed by Rp-HPLC. Chromatographic separation was carried out using a gradient of 20–65% acetonitrile at a flow rate of 0.2 mL/min. (**B**) Overlapped chromatographic profile of samples. (**C**) Area under the peaks 1 and 2 of different samples recorded in tabular form.

The fibrinogenolytic activity of erythrofibrase was further assessed by reverse phase chromatography. The Rp-HPLC profile of untreated fibrinogen showed the presence of two prominent peaks, labelled 1 and 2 (Figure 5A). Fibrinogen treated with thrombin also showed peak 1 and 2 with the same retention time and intensity (Figure 5B,C). However, upon treatment of fibrinogen with erythrofibrase, the intensity of peak 1 was quantitatively reduced (Figure 5C), suggesting probable degradation of proteins present in peak 1, while peak 2 remained unchanged (Figure 5B,C). Further, the Rp-HPLC profile of fibrinogen treated with plasmin showed the presence of only peak 2, indicating complete degradation of the proteins present in peak 1. Cumulative results of the SDS-PAGE profile and Rp-HPLC profile suggest Aα chain degrading activity of erythrofibrase, which shows more similarity with plasmin. Since the protein was purified from *Trimeresurus erythrurus* venom and showed α-fibrinogenase activity, it was named **erythrofibrase.**

**Figure 6:**
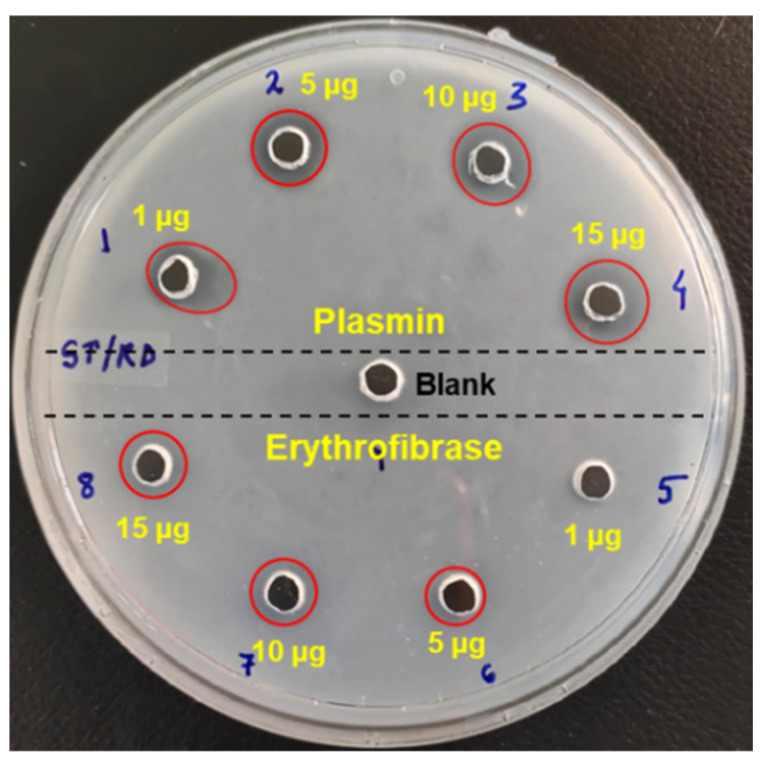
Fibrinolytic activity of erythrofibrase. Fibrin plate was prepared with fibrinogen (5 mg/mL) and thrombin (3 u/mL). Red colour circles around the wells represent zone of clearance.

Fibrinolytic activity was assessed by recording the zone of clearance in the fibrin plate incubated with erythrofibrase. Appearance of a small zone of clearance around the wells (marked by red circles) containing 10 µg and 15 µg of erythrofibrase suggests the mild fibrinolytic activity of erythrofibrase (Figure 6).

**Figure 7:**
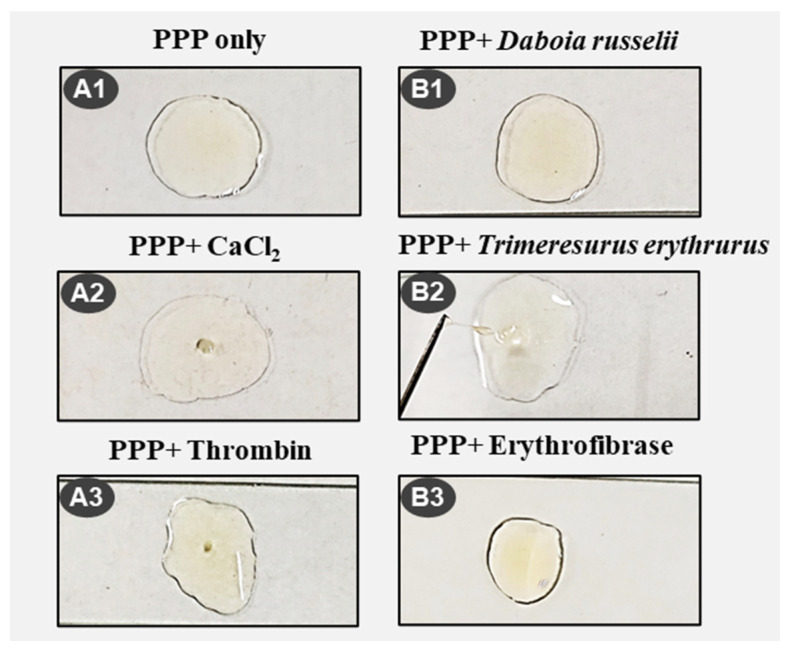
Plasma clot formation and dissolution property of erythrofibrase. Coagulation pattern of platelet-poor plasma (PPP) upon treatment with crude venoms and purified protein. (**A1**–**A3**) represents control set, and (**B1**–**B3**) represents treatment set of experiment. Coagulation time and nature of clot formed were recorded for each experiment using a stop watch.

Since erythrofibrase possesses fibrinolytic activity, its effect on formation and dissolution of a plasma clot was evaluated. The coagulation pattern of platelet-poor plasma (PPP) induced by various treatments were assessed by manual observation of clotting (Figure 7 and Table 1). Platelet-poor plasma (PPP) incubated with CaCl_2_ (normal clotting time) and thrombin resulted in the formation of a solid stable thrombus after 7 min and 3 min, respectively (Figure 7(A2,A3)). Further, no clot formation was observed in plasma treated with crude *Daboia russelii* venom (Figure 7(B1)). However, treatment with crude *Trimeresurus erythrurus* venom resulted in the continuous formation of a jelly-like unstable clot, which was dissolved readily (Figure 7(B2)). Similarly, treatment of plasma with erythrofibrase resulted in the formation of a soft jelly-like thread, which was dissolved within 5 s of formation, with no subsequent clot formation.

### 2.5. Immuno-Reactivity of Erythrofibrase with Antivenom 

**Figure 8:**
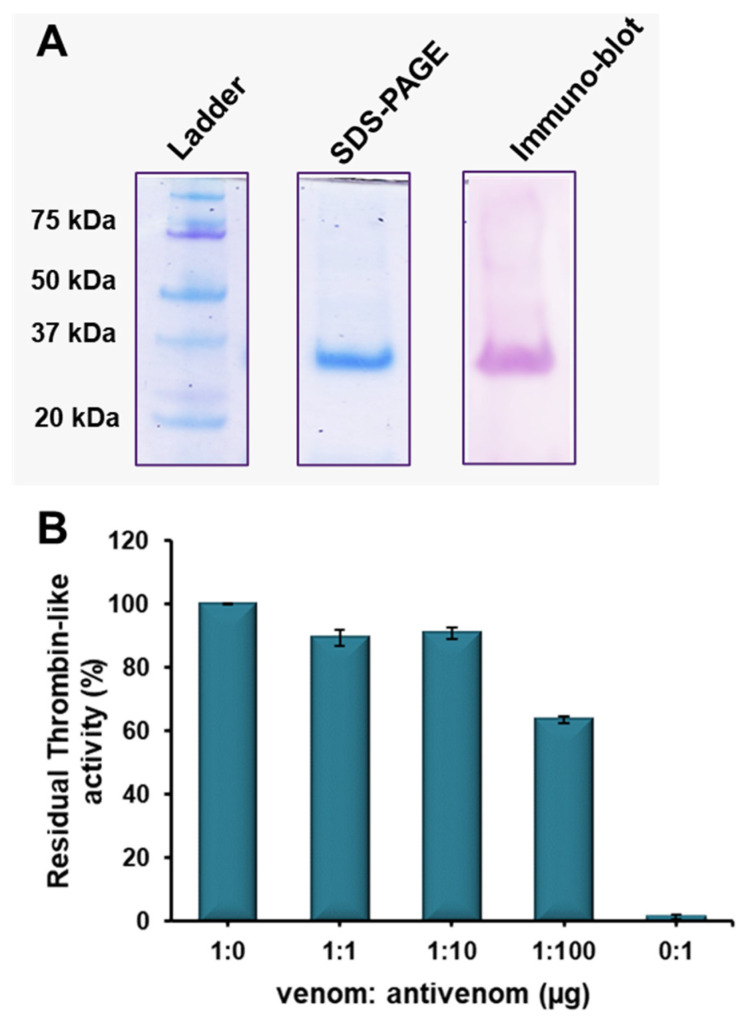

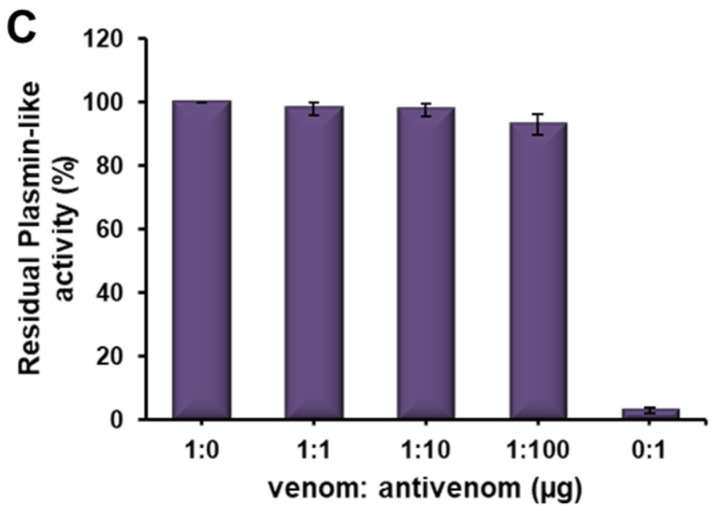
Immuno-reactivity of erythrofibrase with Indian polyvalent antivenom. (**A**) Immuno-blotting of erythrofibrase. Western blotting of erythrofibrase was performed using Indian polyvalent antivenom as primary antibody. (**B**) Neutralization of thrombin-like (**C**) Neutralization of plasmin-like activity. Erythrofibrase (1 µg) and Premium serum polyvalent antivenom was incubated for 1 h at 37 °C in different ratios, followed by respective enzymatic assays. Each data point represents mean ± SD of three independent experiments.

The immuno-recognition of erythrofibrase with Indian polyvalent antivenom was evaluated by Western blotting. The polyvalent antivenom showed complete recognition of erythrofibrase in Western blotting (Figure 8A). The immuno-reactivity of erythrofibrase was further assessed by neutralization experiments. At a ratio of 1:100 (venom/antivenom), Indian polyvalent antivenom showed 35% inhibition of the thrombin-like activity (65% residual activity) of erythrofibrase (Figure 8B). However, polyvalent antivenom did not show any neutralization of the plasmin-like activity of erythrofibrase (Figure 8C), even at higher dose, indicating reduced efficacy of polyvalent antivenom in neutralizing the toxic activities of erythrofibrase. 

## 3. Discussion

Thrombin and plasmin are the key components of the haemostatic system targeting fibrin polymerization and fibrinolysis, respectively, to maintain normal haemostasis. Green-pit-viper-envenomated patients show abnormal fibrinogen and fibrin expanse in the blood which might be associated with the peculiar functioning of venom proteins targeting fibrinogenolytic and fibrinolytic systems [18]. As such, the purification of a haemostatically active protein was undertaken to understand its effect on coagulopathy. Erythrofibrase was purified to homogeneity from *Trimeresurus erythrurus* venom after a two-step reverse phase chromatography showing a single prominent band at ~30 kDa in the SDS-PAGE profile. In-solution trypsin digestion of erythrofibrase and LC-MS/MS analysis of generated peptide fragments resulted in the identification of a protein showing similarity with Alpha-fibrinogenase albofibrase, with a molecular weight of 28 kDa. Albofibrase is a thrombin-like serine protease composed of 258 amino acids, which was previously cloned from a venom gland cDNA library of *Trimeresurus albolabris* [19]. In a subsequent study, albofibrase was expressed in the *Pichia pastoris* system, and the recombinant albofibrase protein showed molecular weight of 30 kDa with 2.2 kDa glycosylation at the 20th residue (NxT glycosylation site) [20]. Erythrofibrase isolated from *T. erythrurus* also showed high similarity in molecular weight and peptide fragments and, therefore, was further characterized. Erythrofibrase showed dose-dependent amidolytic activity on chromogenic substrates S2238 (H-D-Phe-Pip-Arg-*p*NA) along with S2251 (H-D-Val-Leu-Lys-*p*NA), suggesting their thrombin-like and plasmin-like activity. However, erythrofibrase did not alter the recalcification time of platelet-poor plasma, indicating no effect on Ca^2+^ induced coagulation of plasma. Further, erythrofibrase showed mild activity in plasminogen activation assay, which is often observed in thrombin-like enzymes [20].

Snake venom thrombin-like enzymes are named after serine protease thrombin because of their functional similarities [21]. Thrombin is a multifunctional enzyme which plays a central role in the functioning of haemostasis [22]. However, its primary function is fibrin polymerization and clot formation at the end of secondary haemostasis. It is the last proteolytically activated enzyme of the coagulation cascade, which cleaves the Aα and Bβ chain of fibrinogen, releasing fibrin monomers and fibrinopeptides A (FpA) and B (FpB) [22,23]. The fibrin monomers spontaneously polymerize over the loose platelet plug to form a thrombus. Thrombin also activates factor XIII, which further aids in the cross-linking of fibrin monomers, thereby stabilizing the clot [23]. Snake venom thrombin-like enzymes (SVTLEs) are serine endopeptidase which also cleave Arg-Lys bond releasing FpA or FpB (or sometimes both) from the fibrinogen; however, unlike thrombin, they do not usually activate factor XIII [2,4,24]. The fibrin clot formed as a result is not stabilized and, therefore, is readily dissolved by the endogenous fibrinolytic system [4,25]. Such thrombin-like enzymes show high fibrinogen-clotting activity in vitro. Some examples of fibrinogen-clotting enzymes isolated from green pit vipers include albolabrase, purpurase and stejnobin [26,27,28]. The fibrinogenolytic activity of erythrofibrase on bovine fibrinogen was assessed, and it was observed that erythrofibrase could completely degrade the Aα band of bovine fibrinogen (at 48 h incubation) without affecting Bβ and γ chains, indicating it to be an **α-fibrinogenase enzyme**. However, the slow fibrinogenolytic activity of erythrofibrase (activity shown at 48 h) might be due to a partial loss of enzyme activity over time. As an organic solvent is used in reverse-phase chromatography for its purification, this might lead to a partial loss of enzyme activity over time. Fibrinogenases are direct fibrinogenolytic enzymes which degrade fibrinogen into small fragments, rendering it unclottable by thrombin [29]. Unlike fibrinogen-clotting enzymes, they do not convert fibrinogen to fibrin; instead, they decrease the coagulability of fibrinogen. Most of the isolated fibrinogenases are α-fibrinogenase, with only a few that preferentially cleave Bβ chains. Stejnefibrase 1 and 3 (isolated from *Viridovipera stejnegeri*) and alpha-fibrinogenase (isolated from *Calloselasma rhodostoma*) are a few known α-fibrinogenases, while stejnefibrase 2 is a β-fibrinogenase [29,30]. Recombinant albofibrase also shows α-fibrinogenolytic activity, along with showing plasminogen activation similar to erythrofibrase [20]. Moreover, recombinant chitibrisin, GPV-TL1 and TA-2 shows fibrinogenolytic activity on both Aα and Bβ chains of plasminogen-free human fibrinogen, along with showing a variable extent of fibrinogen-clotting activity [31,32,33]. The Aα band-degradation activity of erythrofibrase is comparable to that of the band degradation pattern of plasmin, however, with very low potency. Further, the effect of erythrofibrase on the fibrinolytic system is checked by its fibrin clot dissolution ability, which shows the mild fibrinolytic activity of erythrofibrase. 

The effect of erythrofibrase on plasma clotting was evaluated by manual observation of clot formation. Unlike the stable thrombus formation in the case of thrombin treatment, erythrofibrase showed an initial formation of a thread-like structure which was dissolved within 5 s with no subsequent clot formation. This could be correlated with the fibrinogenolytic activity of erythrofibrase which causes depletion of fibrin monomers, as a result of which clot formation could not be completed. On the other hand, crude venom showed repeated formation and dissolution of jelly-like unstable clots, indicating the presence of fibrinogen-clotting proteins in the venom. Fibrinogen-clotting enzymes cause quick conversion of fibrinogen into unstable fibrin clots [25]. The process of clot formation and dissolution continues until all the fibrinogen is consumed in the process, leading to consumptive coagulopathy [34]. Fibrinogenolytic enzymes like erythrofibrase might be responsible for further acceleration of fibrinogen depletion, thereby aiding in hypofibrinogenemia primarily caused by fibrinogen-clotting enzymes present in crude venom. Thus, consumptive coagulopathy and hypofibrinogenemia together can be suggested to cause the defibrination syndrome and incoagulable blood observed in the *Trimeresurus erythrurus* envenomated patients. 

**Figure 9:**
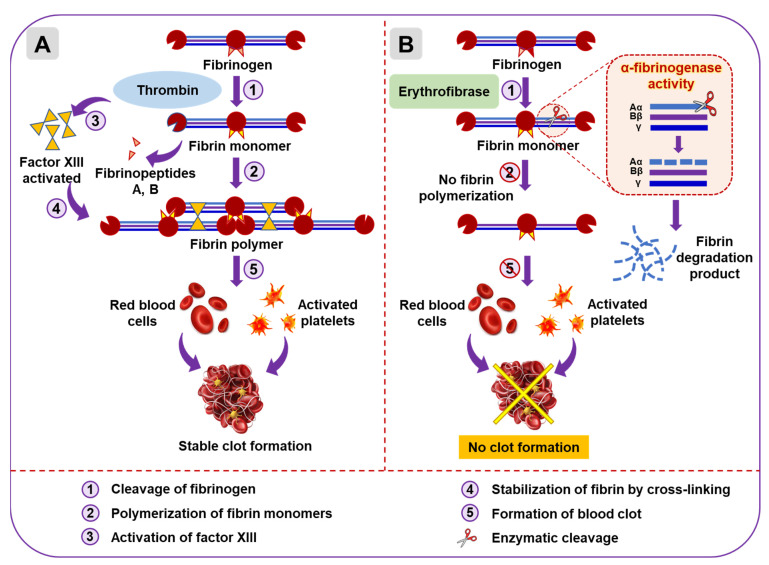
Schematic model showing alpha-fibrinogenase activity of erythrofibrase. (**A**) Cascade of reaction showing thrombin-mediated fibrinogen cleavage and clot formation under normal physiological condition. Thrombin cleaves fibrinogen releasing fibrin monomers, which cross-link over platelet plug and RBCs to form blood clot. (**B**) Effect of erythrofibrase on fibrinogen cleavage and clot formation. Erythrofibrase digests Aα band of fibrinogen, leaving it unavailable for fibrin polymerization and clot formation, thereby rendering the blood incoagulable.

Based on the above experimental outcomes, a schematic model was proposed for erythrofibrase-mediated fibrinogen digestion and its subsequent effect on clot formation (Figure 9).

The envenomation-mediated pathology of green pit vipers cannot be reversed with the administration of currently available Indian polyvalent antivenom [14]. Indian polyvalent antivenom is raised against the venom pool containing a mixture of Big-four venoms. Therefore, it is unlikely for the antivenom to precisely be able to neutralize the venom toxicity of a phylogenetically distant genus such as *Trimeresurus*. The inefficient neutralization potential of the toxic activities of erythrofibrase endorses such inefficacy of Indian polyvalent antivenom. However, Western blotting studies show complete immuno-recognition of erythrofibrase by antivenom. This suggests the presence of immunogenic epitopes, structurally similar to some portion of the erythrofibrase molecule, in the venom pool used to raise the antivenom. The discrepancy in immuno-blotting and neutralization studies might be accounted for by the presence of multiple pharmacological sites in a single molecule, which might be immunogenic to a varying extent. The presence of multiple pharmacological sites in Viperidae venom PLA_2_ molecules, other than the catalytic site, has been reported previously [35]. An antibody’s ability to bind to several antigens (immunogenic epitopes) having confirmational similarities has been established previously; however, not all such binding leads to cross-neutralization of the functional activity of the antigen [36]. Such immunogenic pharmacological sites/epitopes, although they can be recognized with a specific antibody, do not interfere with the catalytic activity of the molecule. This can also be attributed to the large size of the molecules, which provides more contact surface for antibodies to bind without affecting the catalytic site. Moreover, green pit viper monovalent antivenom raised against *Trimeresurus albolabris* is used for treatment of patients in many south-east Asian countries such as Thailand, Vietnam, Malaysia, China, Indonesia, etc. Assessment of the cross-neutralization potential of such con-generic antivenom against the toxic activity of erythrofibrase might provide a better idea of the practical implications of production/import of green pit viper monovalent or region-specific polyvalent antivenom. 

Structurally, snake venom thrombin-like enzymes (SVTLEs) are serine endopeptidases that belong to the trypsin-like serine protease superfamily and peptidase family S1 [37]. They have a typical β/β hydrolase fold, which consists of two β-barrels similar to S1 peptidase serine protease. Each β-barrel contains six beta strands with the insertion of short beta strands and α-helices [4]. The overall three-dimensional structure is divided in two sub-domains N and C, both of which consist of helices and beta strands. The catalytic cleft lies at the junction of two beta barrels (N and C domain), and it is surrounded by various loops. The active site residues, His67, Asp102 and Ser195, are conserved in all S1 family proteases [5]. Apart from the active site, subsites S1 to S3 containing residues Asp189, Gly216 and Gly/Ala226 (chymotrypsin numbering system) are conserved in all SVTLEs which additionally binds to the substrate. Despite the name, thrombin-like enzymes show low structural similarity with thrombin (29–36%) and with other SVTLEs (57–85%) [4]. Unlike three disulfide bridges in thrombin, the presence of six disulfide bonds contributes to stabilize the native structure of the SVTLEs [4]. Thrombin also possesses an additional three-residue S1 loop which allows a substrate with larger side chains to access the catalytic cleft, a feature that is absent in SVTLEs [4]. Further, the shorter length of the 60 loop in SVTLEs leads to the formation of a shallower groove, unlike the deeper groove in thrombin which facilitates substrate binding in the latter. Multiple sequence alignment of albofibrase shows 27.5% similarity with thrombin and 77% similarities with other SVTLEs. Regardless of having high sequence similarity, large functional variation exists among SVTLEs, which are categorized into groups A, B and AB [5,24]. The functional diversity between SVTLEs and thrombin and within SVTLEs is attributed to the variation in amino acid composition, charge distribution and length of the loops surrounding the active site, providing diverse substrate specificity [19,38,39]. Because of their conserved structure and functional diversity, SVTLEs have been considered a potential candidate for therapeutic molecules. They are widely used as defibrinogenating agents and anticoagulants for treating patients with thrombosis, cerebral and myocardial infarction, peripheral vascular diseases and acute ischemia [24]. The commercially available SVTLEs are Arvin^®^ from *Calloselasma rhodostoma* and Defribrase^®^ from *Bothrops atrox* [2].

## 4. Conclusions

The purified protein erythrofibrase is an alpha-fibrinogenase, a toxic component of *Trimeresurus erythrurus* venom that targets the haemostatic system of its prey. Erythrofibrase acts on fibrinogen to cleave the Aα chain, causing substantial depletion in coagulable fibrin monomers. The depletion of coagulable fibrinogen along with consumptive coagulopathy caused by fibrinogen-clotting enzymes results in hypofibrinogenemia leading to incoagulable blood in snakebite patients, which cannot be treated with Indian polyvalent antivenom. This suggests an urgent need for an alternative of current antivenom therapy for green pit viper envenomation, such as a region-specific polyvalent or green pit viper monovalent antivenom.

## 5. Materials and Methodology

### 5.1. Venom and Antivenom

Crude venom of *Trimeresurus erythrurus* was collected with due permission from Chief Wildlife Warden, Environment, Forest and Climate Change department, Mizoram vide 33011/5/5/2011-CWLW/305 dated 18/07/2016 from Aizawl, Mizoram. Post milking, the venom was lyophilised and stored at −20 °C until further use. Lyophilised venom samples were resuspended in Milli-Q water at the time of use, and protein concentration (mg/mL) was determined spectrophotometrically using a Nanodrop 2000 (Thermo Fischer Scientific, Waltham, MA, USA). Indian snake Venom Antiserum (Premium Serum Polyvalent Antivenom, PSPAV) manufactured by Premium Serums and Vaccines Pvt. Ltd., Junnar, Maharashtra, India (Batch no-212013; Exp- 08/2020), a polyvalent antivenom raised against the “Big-four” snake venom (viz. *Naja naja*, *Echis carinatus*, *Daboia russelii* and *Bungarus caeruleus*) was used for immuno-reactivity assessment.

### 5.2. Purification of Erythrofibrase

Crude venom of *Trimeresurus erythrurus* was firstly fractionated using reverse phase chromatography as previously described [17], using Symmetry C18 column (particle size 3.5 μm, a pore size 300 Å) with a linear gradient of 20−65% and 65–80% acetonitrile containing 0.1% (*v*/*v*) TFA for 20−100 min and 100−110 min, respectively. The elution was carried out at a flow rate of 0.8 mL/min at 215 nm, and fraction 7, showing highest amidolytic activity on substrates specific for thrombin (S2238, Chromgenix, Bedford, MA, USA) and plasmin (S2251, Chromgenix, Bedford, MA, USA), was collected. Erythrofibrase was obtained by re-chromatography of fraction 7 on Acclaim^TM^300 C18 reverse phase column (particle size 3 µm, pore size 300 Å) by a linear gradient of 50–65% solvent B (80% acetonitrile containing 0.1% (*v*/*v*) TFA) for 50 min. The elution was carried out maintaining a flow rate of 0.2 mL/min at 215 nm. The fraction was collected manually, and the homogeneity of the collected peak was checked on 12.5% tris-glycine SDS-PAGE in both non-reducing and reducing conditions. 

### 5.3. LC-MS/MS of Purified Protein

Mass spectrometry of the purified protein was performed as previously described by Thakur et al. [17], according to the method given by Kinter and Sherman [40]. The purified Rp-HPLC peak was firstly reduced by DTT in urea (prepared freshly in 50 mM Tris-HCl, pH 8.0) and subjected to in-solution trypsin digestion. The digested peptides in the mixture were separated by desalting using a Nanospray capillary column (PepMapTM RSLC C18, ThermoFisher Scientific, Waltham, MA, USA) and subjected to sequencing by Q-Exactive mass spectrometer (ThermoFisher Scientific, Waltham, MA, USA). The raw files were viewed in Proteome Discoverer 2.2 (ThermoFisher Scientific, Waltham, MA, USA) using the Sequest program. Double and triple charged peptides were selected and, based on sequence similarity, peptide fragments were assigned to proteins in the NCBI database. The peptide fragments generated by MS/MS were aligned with the identified protein sequence retrieved from the NCBI database manually. 

### 5.4. Multiple Sequence Alignment

Proteins showing high similarity with erythrofibrase were identified by the online blastp algorithm (http://blast.ncbi.nlm.nih.gov), accessed on 3 July 2022 and their FASTA sequence was retrieved from the NCBI database. Multiple sequence alignment of the partial sequence of erythrofibrase obtained by MS/MS and sequences of other thrombin-like proteins was performed using online Clustal Omega software (https://www.ebi.ac.uk/Tools/msa/clustalo/). Residues were highlighted in different colours manually. 

### 5.5. Functional Characterization of Erythrofibrase

#### 5.5.1. Amidolytic Activity Assay 

Amidolytic activity of erythrofibrase was assessed on chromogenic substrates S2238 and S2251 (Chromogenix, Bedford, MA, USA), which are specific for thrombin and plasmin, respectively. Briefly, different doses of erythrofibrase (0.01–10 µg/mL) were preincubated with assay buffer (20 mM Tris-Cl, 150 mM NaCl and 1% BSA, pH 7.4) for 2 min at 37 °C followed by the addition of 50 µL of substrate to initiate the reaction. Change in absorbance was monitored at 405 nm for 10 min using a MultiSkan GO Spectrophotometer (ThermoFisher Scientific, Waltham, MA USA). Crude venom (10 µg/mL) was used as positive control. Enzyme activity was expressed in µmoles of substrate hydrolysed per minute (µmol/min). 

#### 5.5.2. Clotting Assay

The effect of erythrofibrase on coagulation of platelet-poor plasma (PPP) was measured by calculating recalcification time (RT) as described by Deka et al. [41]. Different doses (0.01–10 µg/mL) of erythrofibrase were preincubated with PPP for 2 min at 37 °C. Clot formation was initiated by addition of 50 mM CaCl_2_, and change in OD was monitored at 405 nm every 10 s for 900 s using a MultiSkan GO Spectrophotometer (ThermoFisher Scientific, Waltham, MA, USA). The clotting time of PPP with assay buffer was considered the normal clotting time (NCT), and the clotting time of crude venom (10 µg/mL) was used as a positive control.

#### 5.5.3. Plasminogen Activation Assay 

Plasminogen activation assay of erythrofibrase was performed according to the method used by Park et al. [42]. Briefly, different doses of erythrofibrase (0.1–10 µg/mL) were incubated with 0.01 U of plasminogen in a total volume of 100 µL of assay buffer (20 mM Tris-Cl buffer and 150 nM NaCl, pH 7.5) for 10 min at 37 °C. Following incubation, 20 µL aliquot was mixed with 180 µL of plasmin-specific substrate S2251 (1 mM stock). The release of coloured product *p*-nitroaniline (*p*-NA) was monitored by measuring OD at 405 nm for 15 min. Ten nanomolar of tissue-plasminogen activator (t-PA) was taken as positive control. One unit of plasminogen activation (U) was expressed as micromoles of substrate hydrolysed per minute.

#### 5.5.4. Fibrinogenolytic Activity 

The fibrinogenolytic activity of erythrofibrase was estimated by an electrophoretic method in both a dose-dependent and time-dependent manner. Firstly, different doses of erythrofibrase (0.1–10 µg) were preincubated with 50 µL bovine plasma fibrinogen (2 mg/mL) and 50 µL assay buffer (50 mM Tris-Cl, 150 mM NaCl, pH 7.4) for 1 h at 37 °C. Following incubation, the resultant products were subjected to SDS-PAGE using 10% tris-glycine gel in reducing condition. For time-dependent analysis, a fixed dose of erythrofibrase (10 µg) was incubated with fibrinogen in similar conditions to those mentioned above. Aliquots were taken at different time intervals ranging from 0 min to 48 h and subjected to electrophoresis. Fibrinogen without venom was considered as a negative control. Fibrinogen treated with thrombin (10 u/mL), plasmin (1 µg) and crude venom (5 µg) was considered as a positive control.

The fibrinogenolytic activity of erythrofibrase was also evaluated by chromatographic method for the analysis of cleavage pattern. Firstly, 10 µg of erythrofibrase was mixed with 100 µg of bovine fibrinogen in 50 mM Tris-Cl, 150 mM NaCl, pH 7.4 and incubated at 37 °C for 1 h. Fibrinogen only and fibrinogen treated with thrombin and plasmin were taken as negative and positive controls for the experiment. Following incubation, the tubes were cooled in a refrigerator for 5 min and subjected to Rp-HPLC using Acclaim C18 column with a linear gradient of 20–65% acetonitrile with 0.01% TFA. Intensity of peaks was calculated by quantifying the area under the peaks (mAu*min) using chromeleon 6.8 software (ThermoFisher Scientific, Waltham, MA, USA)

#### 5.5.5. Fibrinolytic Activity

Fibrinolytic activity of erythrofibrase was assessed according to the method described by Astrup and Mullertz [43] and slightly modified by Kaur et al. in 2019 [44]. Firstly, a fibrin plate was prepared by mixing fibrinogen (5 mg/mL) and thrombin (3 U/mL) in 1% agarose (*w*/*v*). The plate was allowed to stand at room temperature until fibrin polymerization. Wells were punctured post-polymerization, and different doses (1–15 µg) of protein and plasmin were added to the wells. Assay buffer without any treatment was taken as a negative control. The plate was allowed to stand at room temperature for 2 h and then, incubated overnight at 37 °C. The presence of a zone of clearance around the wells confirmed fibrinolytic activity.

#### 5.5.6. Plasma Clot Formation/Dissolution Activity

The coagulation activity of platelet-poor plasma (PPP) upon treatment with crude venoms and erythrofibrase was observed by manual examination of clot formation and dissolution. Firstly, 50 µL of platelet-poor plasma isolated from human blood was mixed with 25 µL of 20 mM Tris-Cl, pH 7.4 on a clean glass slide. Clot formation was initiated upon treatment with CaCl_2_, thrombin, crude *Daboia russelii* venom, crude *Trimeresurus erythrurus* venom and erythrofibrase separately. The formation of a clot was observed by the appearance of fine thread-like structures on the needle. For each set of experiments, the clot formation time, clot dissolution time and nature of clot were recorded. Platelet-poor plasma only was taken as the negative control. 

### 5.6. Immuno-Reactivity of Erythrofibrase with Antivenom

*Western blotting*: Immuno-recognition of erythrofibrase with commercially available Indian polyvalent antivenom was assessed by Western blotting [45]. Erythrofibrase (8 µg) was subjected to 12.5% tris-glycine gel in the reduced condition, and electrophoresis was carried out at 120 V using Mini PROTEAN (Bio-Rad, CA, USA). The gel was then electro-transferred to pre-soaked PVDF membrane (activated by methanol) at 100 volts for 90 min. The membrane was subsequently blocked with 1% BSA in TBST for 1 h at room temperature, followed by washing thoroughly with a wash buffer (TBST). The membrane was incubated overnight at 4 °C with 1:500 (*v*/*v*) dilutions of Indian polyvalent antivenom (10 mg/mL). The membrane was then washed with wash buffer and incubated with 1:1000 (*v*/*v*) dilution of alkaline phosphatase conjugated anti-horse IgG for 2 h with gentle shaking. The membrane was washed again rigorously with the wash buffer and developed using pre-mixed 5-bromo-4-chloro-3-indolyl phosphate (BCIP)/nitro blue tetrazolium (NBT) liquid substrate till colour appeared. The reaction was stopped with 1% acetic acid.

*Neutralization assays*: Immuno-reactivity of erythrofibrase with Indian polyvalent antivenom was further assessed by neutralization studies. Neutralization assays were performed for both thrombin-like activity and plasmin-like activity of erythrofibrase. Erythrofibrase (1 µg) was incubated with an assay buffer containing Indian polyvalent antivenom in a venom/antivenom ratio of 1:0, 1:1, 1:10, 1:100 and 0:1 ratio (*w*/*w*) at 37 °C for 1 h. Post incubation, the mixtures were subjected to thrombin-like activity and plasmin-like activity assays as described earlier. Erythrofibrase incubated with an assay buffer without antivenom was used as positive control for all sets of experiments. Percentage inhibition was calculated considering enzyme activity of the control as 100%.

## Figures and Tables

**Table 1:** Summary of coagulation pattern of platelet-poor plasma (PPP) upon treatment with various components.

Protein Sample	Treatment	Clotting Time	Clot Dissolution Time	Nature of Clot	Image
-	CaCl_2_	7 min	-	Thrombus	A2
*Daboia russelii*	-	No clot	-	No clot	B1
*Daboia russelii*	CaCl_2_	3 min	-	Thrombus	
*Trimeresurus erythrurus*	-	25 s	30 s	Continuous formation and dissolution of jelly like clot	B2
		7 min	15 min		
		19 min	20 min		
		23 min	24.5 min		
*Trimeresurus erythrurus*	CaCl_2_	7 min	-	Thrombus	
Erythrofibrase	-	25 s	30 s	Thin jelly like clot	B3
-	Thrombin	3 min	-	Small stable clot	A3

## Data Availability

Data that support the findings of this study can be available from the corresponding author upon reasonable request.

## Supplementary Materials

The following supporting information can be downloaded at: https://www.mdpi.com/article/10.3390/toxins16040201/s1, Table S1: Peptide sequence of trypsin digested fragments of erythrofibrase obtained by LC-MS/MS analysis.
