# Supplementary material for: Isolation and Functional Characterization of Erythrofibrase: An Alfa-Fibrinogenase Enzyme from Trimeresurus erythrurus Venom of North-East India
Source: Toxins (Basel). 2024 Apr 22;16(4):201. doi: 10.3390/toxins16040201 (PMC11054993; doi:10.3390/toxins16040201)
Supplement: Supplementary file 1 [file toxins-16-00201-s001.zip › toxins-2924808-supplementary.pdf]

Supplementary Materials: Isolation and Functional Characterization of Erythrofibrase: An Alfa-Fibrinogenase Enzyme from *Trimeresurus erythrurus* Venom of North-East India

Susmita Thakur, Rafika Yasmin, Anita Malhotra, H. T. Lalremsanga, Vishal Santra, Surajit Giri and Robin Doley

Table S1. Peptide sequence of trypsin digested fragments of erythrofibrase obtained by LC-MS/MS analysis.

Protein identity	Peptide fragments	Position	No. of peptides	z	MH+ [Da]	Score
Alpha-fibrinogenase albofibrase (P0CJ41)	KLLNEDEQIR	81-90	1	3	1257.68	294.83
	KLLNEDEQIRNPK	81-93	2	3	1597.85	
	LLNEDEQIR	82-90	2	2	1129.58	
	LLNEDEQIRNPK	82-93	2	3	1468.78	
	LLNEDEQIRNPKEK	82-95	1	4	1725.91	
	EKFICPNK	94-101	1	2	4344.26	
	KSNEILDKDIMLIK	102-115	2	4	1675.93	
	SNEILDK	103-109	1	2	2764.27	
	SNEILDKDIMLIK	103-115	2	2	1531.84	
	SNEILDKDIMLIKLDSPVSNSA-HIAPLSLPSSPPSVGSVCR	103-143	2	5	4344.26	
	DIMLIK	110-115	2	1	732.43	
	DIMLIKLDSPVSNSAHIAPLSLPSSPPSVGSVCR	110-143	2	4	3544.85	
	LDSPVSNSAHIAPLSLPSSPPSVGSVCR	116-143	2	3	2831.44	
	IMGWGSTTPIEVTYPDVPYCANINLLDDAECK-PGYPELLPEYR	144-187	3	4	4957.34	
	TLCAGIVQGGK	188-198	2	1	1103.59	
	TLCAGIVQGGKDTCGGDSGGPLICNEK	188-214	2	2	2764.27	
	DTCGGDSGGPLICNEK	199-214	2	2	1679.7	
	LHGIVSYGGHPCGQSHKPGIYTNVFDYNDWIQSI-IAGNTDATCLS	214-248	1	5	4963.32	
